# Hyperthermia enhances 17-DMAG efficacy in hepatocellular carcinoma cells with aggravated DNA damage and impaired G2/M transition

**DOI:** 10.1038/srep38072

**Published:** 2016-12-02

**Authors:** Zhizhou Huang, Xueqiong Zhou, Yangfan He, Xiangyu Ke, Ying Wen, Fei Zou, Xuemei Chen

**Affiliations:** 1 Department of Occupational Health and Medicine, School of Public Health, Southern Medical University, 1838 Guangzhou Road North, Guangzhou, 510515, China

## Abstract

Due to the lack of effective treatment, hepatocellular carcinoma (HCC) is one of the malignancies with low survival rates worldwide. Combination of hyperthermia and chemotherapy has shown promising results in several abdominal tumours, but high expression of HSP90 in tumours attenuated the efficacy of hyperthermia. Thus a combination of hyperthermia and inhibition of HSP90 might be a feasible therapeutic strategy for HCC. One hepatic cell line (L02) and two HCC cell lines (Huh7 and HepG2) were heated at 42 °C for 0, 0.5 or 4 h with or without 100 nM 17-dimethylaminoethylamino-17-demethoxygeldanamycin (17-DMAG). HCC cells of the combination group exhibited more G2/M arrest and higher apoptotic rates which might result from suffering from more reactive oxygen species and serious DNA damage. Heat shock/17-DMAG co-treatment of HCC cells also destabilized CDK1, Cyclin B1 and CDC25C with a concomitant decreased proportion of cells in the M phase. Furthermore, co-treatment impaired the interaction of HSP90α with CDC37 and with CDK1, accompanied with decreased soluble CDK1. Combination of 17-DMAG with a 1.5-h whole body hyperthermia treatment attenuated tumour growth in xenograft mice models. These results suggest hyperthermia sensitize HCC to 17-DMAG, and combination of hyperthermia with 17-DMAG might be a potential therapeutic strategy for HCC.

Liver cancer, which is one of the most common malignancies in the world, ranked second among cancer-related causes of death in men of worldwide in 2012^1^. Hepatocellular carcinoma (HCC) constitutes over 90% of primary liver cancers^2^. One of the reasons for the high mortality rate is that a majority of patients are asymptomatic in the early stages of HCC and symptoms often only occur at an advanced stage. Another reason is the lack of an effective treatment for HCC^3^. Currently, the main therapeutic methods for HCC include surgical resection, radiofrequency ablation, transarterial chemoembolization and transplantation. With the exception of transplantation, these therapeutic approaches bring only little benefit to the patients, as reflected by high recurrent rates and low survival rates^4^,^5^. Thus, it is urgent to explore a more effective therapy for HCC.

Hyperthermia, raising the temperature of a tumour to 40–45 °C^6^, is being used more and more widely in abdominal tumours as an adjuvant therapy in combination with chemotherapy, named hyperthermic intraperitoneal chemotherapy (HIPEC), after cytoreductive surgery. HIPEC has been performed in several abdominal tumour entities, such as ovarian cancer, colorectal cancer and gastric cancer, and successfully prolonged long-term survival^7^,^8^,^9^. Whether a combination of hyperthermia and chemotherapy would be beneficial for survival of liver cancer patients is not known. Unfortunately, patients with high peritoneal tumour burden are less responsive to co-treatment with hyperthermia and chemotherapy^10^, results in reducing the efficacy below expectations. Hence, the combination of other forms of hyperthermia, such as regional hyperthermia and whole body hyperthermia, are in the on-going clinical trial^11^. Besides hyperthermia forms, the other possible reason is that tumour cells express more heat shock proteins (HSPs), including HSP90, HSP70 and HSP27^12^, which induces general stress resistance and promotes tumour cells survival during heat stress.

Among the HSPs, HSP90, an ATP-dependent molecular chaperone, was shown to be very important for hepatocarcinogenesis and HCC cell survival in stress conditions^13^. HSP90 includes two major isoforms—HSP90α (inducible under stress) and HSP90β (constitutively expressed). HSP90 can stabilize and regulate its substrate proteins (also called clients) including transcription factors, kinases and steroid hormone receptors^13^. Many of the kinases implicated in pathogenesis of HCC, such as kinases in the phosphatidylinositol-3 kinase (PI3K)/AKT and RAF/MEK/ERK pathways, which promote cell survival and cell proliferation, respectively^3^, are HSP90 clients (http://www.picard.ch/downloads/HSP90interactors.pdf). Tumour cells generally express HSP90 at higher levels than normal tissue^14^ and high expression of HSP90 is associated with poor prognosis and a poor overall survival rate^15^. Moreover, co-chaperones of HSP90, including HSP70, HOP, CDC37, CHIP, p23, which regulate HSP90 ATPase activity, help to recognize its clients, and facilitate final maturation or degradation of clients^16^, are also implicated in cancer cell survival. For example, HSP90 and CDC37 form a complex to stabilize kinases of multiple signalling pathways, some of which are involved in carcinogenesis^17^.

In this study, we explored whether hyperthermia could enhance the anti-tumour effect of the HSP90 inhibitor 17-DMAG in HCC, and demonstrated that the increased therapeutic benefit of this new strategy in HCC.

## Results

### 17-DMAG/hyperthermia co-treatment inhibits HCC growth *in vivo* with decreased CDK1, Cyclin B1 level

**Figure 1:**
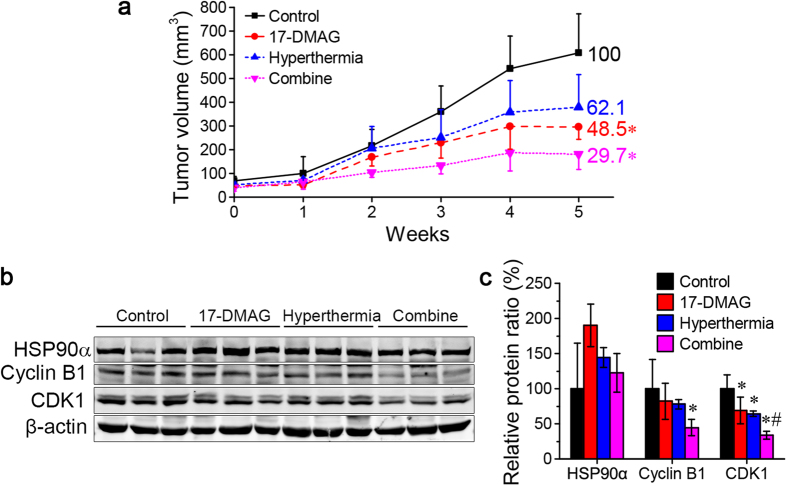
Hyperthermia/17-DMAG combination treatment significantly reduced growth of xenograft tumours in nude mice. 5 × 10^6^ Huh7 cells were injected into 5-week old male BALB/c nude mice. 10 days post injection mice with xenograft tumours were treated with a 1.5-h heat shock at 42 °C and/or 25 mg/kg 17-DMAG three times a week. (**a**) Growth curves of xenograft tumours treated with 17-DMAG or heat shock alone or in combination (n = 5). Co-treatment with 17-DMAG and heat shock reduced tumour growth the most. (**b**) After the final treatment, animals were sacrificed and Hsp90α, Cyclin B1 and CDK1 in 3 tumours of each group were detected by Western blot. (**c**) The bands were quantified with Image J revealing a significant decrease of Cyclin B1 and CDK1 after co-treatment. Results are presented as mean ± SD. *P < 0.05 vs. control group; ^#^*P* < 0.05 vs. 17-DMAG group.

To explore the effect of co-treatment of hyperthermia and 17-DMAG *in vivo*, HCC cell lines Huh7 cells were injected into nude mice. Xenograft tumour growth curves are shown in Fig. 1a and images of tumours are shown in Fig. S1. In the combination group, the mean tumour volume was the smallest from the 2^nd^ week until the end. At the end of the treatment, cell cycle regulation protein CDK1 and Cyclin B1 were detected by Western Blot (Fig. 1b). Expressions of CDK1 and Cyclin B1 in the combination group were significantly lower than the control group (Fig. 1c). The result showed hyperthermia sensitized HCC to 17-DMAG *in vivo*, and which might correlate with cell cycle regulation and the changing of the CDK1-Cyclin B1 complex.

### Combination of heat shock and 17-DMAG compromised cell viability with G2/M arrest

**Figure 2:**
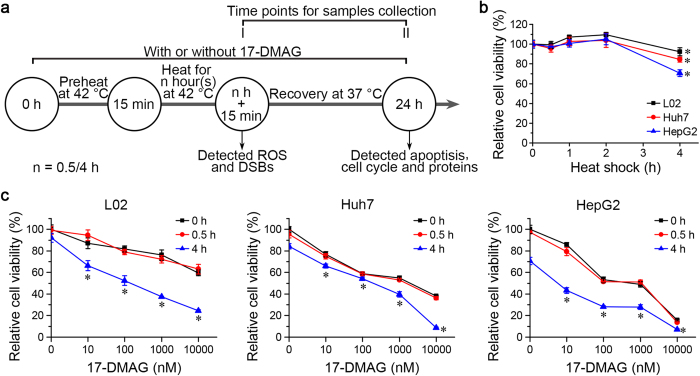
Heat shock sensitizes HCC cells to 17-DMAG. L02, Huh7 and HepG2 cells were heated for the indicated time in the absence or presence of 17-DMAG at 42 °C, then recovered at 37 °C until 24 h after the start of the experiment. Cell viability was detected by CCK-8 assay. (**a**) The scheme of cell treatment and sample collection. (**b**) Changes of cell viability after heat shock alone. (**c**) Changes of cell viability after co-treatment with heat shock and 17-DMAG. Results are shown as mean ± SD, n = 3. *P < 0.05 vs. 0 h heat shock group.

**Figure 3:**
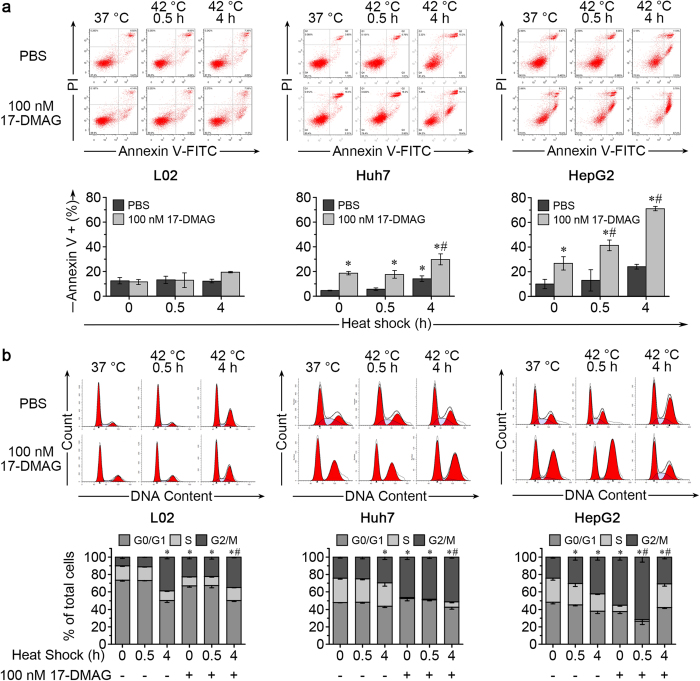
Combination of heat shock and 17-DMAG increased the apoptosis rate and relative frequency of G2/M cell cycle phase in Huh7 and HepG2 cells. L02, Huh7 and HepG2 Cells were heated for the indicated time with or without 17-DMAG at 42 °C, then allowed to recover at 37 °C until 24 h. Flow cytometry was used to monitor the changes of apoptosis rates and cell cycle distribution. (**a**) Images of flow cytometry results (upper panels) and frequency histogram of Annexin V positive cells (lower panels) of L02, Huh7 and HepG2. (**b**) Images of cell cycle results (upper panels) and stacked bar charts (lower panels) of cell cycle phase distribution of L02, Huh7 and HepG2. *P < 0.05 vs. 0 h heat shock PBS group, ^#^*P* < 0.05 vs. 17-DMAG alone group. Results are shown as mean ± SD, n = 3.

To further study the mechanism of the synergistic effect of hyperthermia and Hsp90 inhibitor, L02, Huh7 and HepG2 cells were heated for 0.5, 1, 2 and 4 h at 42 °C with or without addition of 17-DMAG, then allowed to recover at 37 °C until 24 h after the start of the experiment (Fig. 2a). While heat shock for 0.5 to 2 h did not affect cell viability, a 4-h heat shock attenuated cell viability slightly for L02 (92.32 ± 4.18%) but more strongly for HepG2 (70.75 ± 3.41%) (Fig. 2b). When cells were treated with 10 to 10,000 nM 17-DMAG and then heat treated for 0, 0.5 and 4 h at 42 °C with subsequent recovery at 37 °C until 24 h cell viability was compromised more severely, indicating a synergistic effect of the combination treatment (Fig. 2c). HCC cell line QSG-7701 demonstrated attenuated cell viability after 4 h at 42 °C with addition of 17-DMAG, comparing to hepatic cell line QGY-7703. (Fig. S2). The IC50s of 17-DMAG were >10,000 nM, 1279 (95% CI: 737, 2,487) nM and 355 (95% CI: 190, 670) nM for L02, Huh7 and HepG2, respectively. When 17-DMAG was applied in combination with a 4-h heat shock, IC50s decreased to 140 (95% CI: 54, 307) nM, 113 (95% CI: 52, 218) nM and 4 (95% CI: 0.4, 14) nM for L02, Huh7 and HepG2, respectively. The 4-h heat shock combined with 100 nM 17-DMAG significantly increased the number of apoptotic Huh7 and HepG2 cells (Fig. 3a). 17-DMAG treatment increased the proportion of cells in the G2/M cell cycle phase more prominently in Huh7 and HepG2 than in L02 cells, and the combination of heat shock and 17-DMAG treatment further exacerbated the G2/M arrest in Huh7 and HepG2 cells (Fig. 3b).

### Heat shock increased ROS levels and aggravated 17-DMAG induced double strand breaks

**Figure 4:**
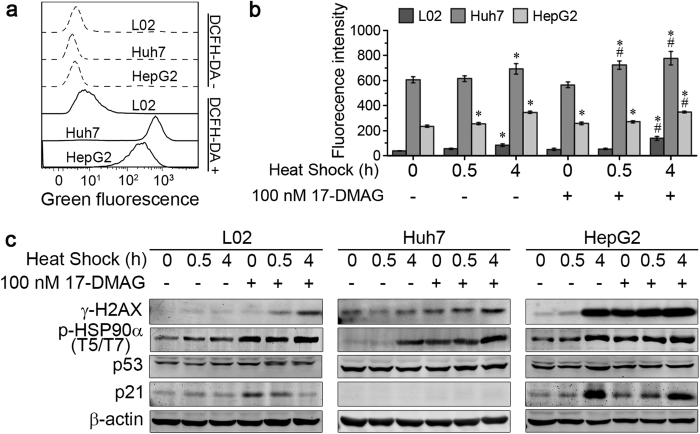
Heat shock increased ROS levels and aggravated 17-DMAG induced DSBs. L02, Huh7 and HepG2 cells were heated for the indicated time with or without 17-DMAG at 42 °C, and ROS levels were detected immediately after heat shock. Then cells were allowed to recover at 37 °C until 24 h, and DSBs related proteins were detected by Western blot. **(a**) HCC cells Huh7 and HepG2 presented higher basal level of ROS than L02. (**b**) ROS level increased after heat shock plus 17-DMAG. (**c**) γ-H2AX and p-HSP90α (T5/T7) increased at 24 h after co-treatment, and p21 increased in L02 and HepG2 but not in p21 defective cell line Huh7. *P < 0.05 vs. untreated control group, ^#^*P* < 0.05 vs. 17-DMAG alone group. Results are shown as mean ± SD, n = 3.

In cells treated with heat shock for 0, 0.5, and 4 h in absence and presence of 17-DMAG, reactive oxygen species (ROS) were determined by flow cytometry immediately after treatment (time point I, Fig. 2a). Both HCC cells, Huh7 and HepG2, had a much higher basal level of ROS than L02 cells (Fig. 4a). In the absence of 17-DMAG a 4-h heat shock induced an increase in ROS production in all 3 cell lines (Fig. 4b). In the presence of 17-DMAG, the ROS production was increased already in the absence of heat shock (HepG2), after 0.5-h heat shock (Huh7) and after a 4-h heat shock (L02, Huh7 and HepG2), respectively. Since ROS induces DNA damage, we quantified proteins associated with the DNA damage and repair response by western blot 24 h after heat shock treatment with or without 17-DMAG (time point II, Fig. 2a). DNA double-strand breaks (DSBs) activate DNA-activated protein kinase (DNA-PK), which is shown to phosphorylate T5 and T7 residues of HSP90α^18^,^19^. Therefore, we also detected p-HSP90α (T5/T7) as an indicator of DNA damage. In all three cell lines a synergistic effect of 17-DMAG and heat shock on γ-H2AX and p-HSP90α (T5/T7) indicating more severe DNA damage through the combination treatment. The DNA damage response-induced G2/M arrest activates p53 through ATM/ATR, which further transactivates p21^20^. In turn, p21 interferes with the activation of CDK1 and thus prevents the activation of the CDK1-Cyclin B1 complex^21^. Therefore, we checked p53 and p21 in the 3 cell lines. Although p53 did not change considerably, p21 in HepG2 increased after co-treatment. Huh7 is a p53 mutant and p21 defective cell line^22^. The p21 status and consequently different CDK1-Cyclin B1 complex activation might contribute to the different response to DNA damage observed in Huh7 and HepG2 cells.

### Heat shock enhanced 17-DMAG induced G2 arrest with decreased Cyclin B1 levels in HCC

**Figure 5:**
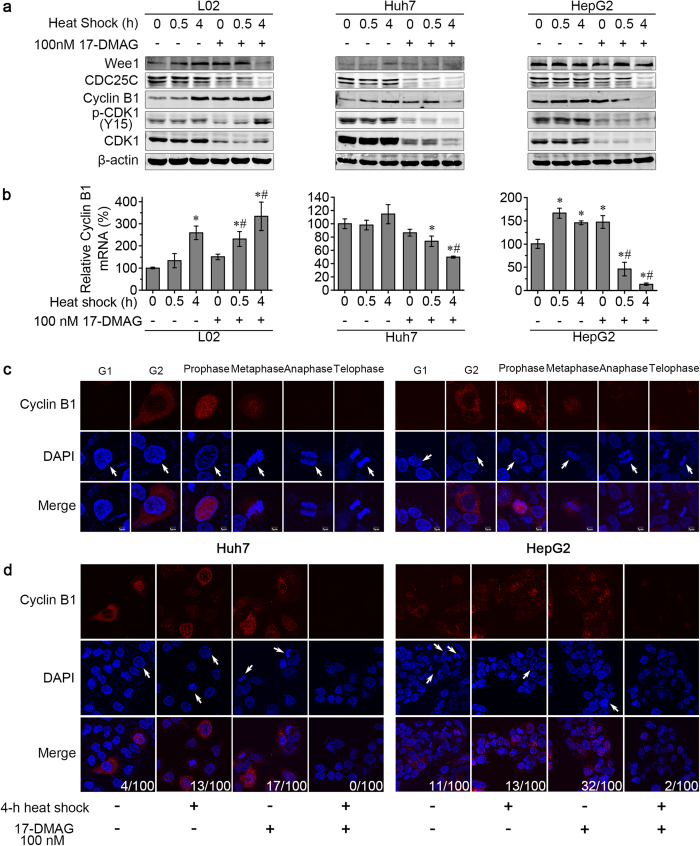
Combination of heat shock and 17-DMAG decreased the level of Cyclin B1 and reduced the frequency of M phase Huh7 and HepG2 cells. L02, Huh7 and HepG2 cells were heated for the indicated time with or without 17-DMAG at 42 °C, then cells were allowed to recover at 37 °C until 24 h. The levels of G2/M-related proteins were detected by Western blot. The levels and location of Cyclin B1 were further confirmed by immunofluorescence staining. (**a**) After co-treatment, Cyclin B1 accumulated in L02 cells, but decreases in Huh7 and HepG2 cells. (**b**) Changes in Cyclin B1 mRNA amount changes in parallel to the protein level. (**c**) The levels and location of Cyclin B1 in Huh7 and HepG2 cells with normal cell cycle transition in control cells. Cell phases were determined by nuclei morphology (arrows). (**d**) After co-treatment, Huh7 and HepG2 cells presented less cells in M phase (indicated by white arrows and counted in 100 cells) and decreased Cyclin B1 levels.

We previously found that 17-DMAG alone induced Cyclin B1 accumulation in HCC cells^23^. We next studied how co-treatment of heat shock and 17-DMAG would influence cell cycle regulator proteins. A 4-h heat shock increased the proportion of cells in G2/M phase in L02 cells but no obvious synergistic effect of heat shock with 17-DMAG was observed (Fig. 3b). In contrast, Huh7 and HepG2 cells were much more sensitive to the HSP90 inhibitor 17-DMAG, as evidenced by the higher G2/M proportions. Furthermore, compared to the treatment with 100 nM 17-DMAG alone, more HepG2 cells arrested in G2/M after co-treatment with 0.5-h heat shock and 17-DMAG (Fig. 3b). To analyse the effects of heat shock and 17-DMAG on the cell cycle in more detail we detected key cell cycle regulator proteins for cell transiting through G2/M phase^24^, such as Wee1, CDC25C, Cyclin B1 and CDK1, by Western blotting. In all three cell lines Wee1, CDC25C and CDK1 decreased after 4-h heat shock and 17-DMAG co-treatment. Interestingly, Cyclin B1 nearly disappeared in Huh7 and HepG2 after the 4-h heat shock in the presence of 17-DMAG, but increased clearly in L02 cells (Fig. 5a). Cyclin B1 mRNA showed the same trends as the changes of protein (Fig. 5b). The decrease of Cyclin B1 was further confirmed by immunofluorescence staining in Huh7 and HepG2 cells (Fig. 5d). Cell phases were determined by nuclei morphology under confocal laser scanning microscope as previous report^25^. Compared to the normal cell cycle in control Huh7 and HepG2 cells (Fig. 5c), the co-treatment of 4-h heat shock with 24-h 17-DMAG remarkably decreased Cyclin B1, and cells in M phase were hardly seen (Fig. 5d). Combined with the results of flow cytometry, these data suggest co-treatment impairs the G2/M transition and arrests HCC cells in early G2 phase with lower cyclin B1 level.

### Heat shock/17-DMAG co-treatment attenuates HSP90-Cdc37 interaction and results in CDK1 aggregation

**Figure 6:**
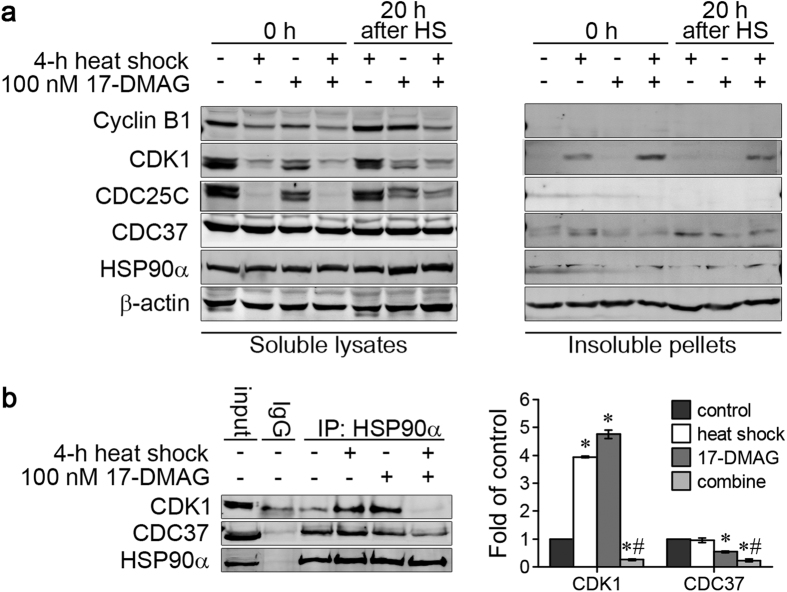
Co-treatment with heat shock and 17-DMAG attenuated the interaction of HSP90 and CDC37 and induced the aggregation of CDK1 in Huh7 cells. (**a**) Huh7 cells were heated for 4 h at 42 °C with or without 100 nM 17-DMAG and then allowed to recover for 20 h at 37 °C. One group of cells was collected immediately after the heat shock (designated as 0 h) and another group after the recovery period (designated as 20 h), and Cyclin B1, CDK1, CDC25C, CDC37 and Hsp90α levels in the soluble lysates and insoluble pellets were detected by Western Blot. Control cells were left untreated. CDK1 was detected in the insoluble fraction directly after HS but not after the recovery period in the absence of 17-DMAG. Co-treatment with heat shock and 17-DMAG increased the aggregation of CDK1 and prevented resolubilization. (**b**) Huh7 cells were heated at 42 °C for 4 h and treated with 100 nM 17-DMAG alone or in combination, then allowed to recover at 37 °C for 20 h. CDK1 and CDC37 were co-immunoprecipitated with HSP90α (left panel), and band intensity were analysed by Image J (right panel). 17-DMAG reduced the amount of coprecipitated CDC37. Though heat shock or 17-DMAG alone increased the interaction of CDK1 and Hsp90α, the co-treatment decreased this interaction (A representative result of 3 independent experiments is shown). *P < 0.05 vs. untreated control group, ^#^*P* < 0.05 vs. 17-DMAG alone group. Results are shown as mean ± SD, n = 3.

Since the G2/M transition depends on the formation of the metaphase promotion factor (MPF), CDK1-Cyclin B1 complex, and since Cyclin B1 decreased in HCC, we analysed the changes in protein stability caused by co-treatment of Huh7 cells with heat shock and 17-DMAG (Fig. 6a). Protein samples were collected either directly after heat shock (time point I, Fig. 2a) or after 20 h recovery at 37 °C (time point II, Fig. 2a). Directly after heat shock CDK1 was found in the insoluble pellets in absence and presence of 17-DMAG. If cells were allowed to recover for 20 h after heat shock, only after combined treatment with heat shock and 17-DMAG CDK1 was still detected in the insoluble fraction (Fig. 6a, the right panel). As many kinases are HSP90 clients and docking to HSP90 needs the assistance of CDC37, we further investigated if the accumulation of insoluble CDK1 is associated with the dynamics of the HSP90α-CDC37 interaction. Huh7 cells were heated for 4 h at 42 °C with or without 100 nM 17-DMAG and subsequently allowed to recover at 37 °C for 20 h. CDK1 and CDC37 were co-immunoprecipitated with HSP90α (Fig. 6b). Though CDC37 did not change clearly after heat shock and 17-DMAG treatment (Fig. 6b), more CDC37 and CDK1 bound to HSP90α after the recovery from heat shock. 17-DMAG attenuated the interaction of CDC37 and HSP90α, but did not affect the interaction of HSP90α and CDK1. A combination of heat shock and 17-DMAG resulted in less CDC37 and little CDK1 binding to HSP90α (Fig. 6b). These results showed that CDK1 tends to aggregate during heat stress, and needs to be stabilized by HSP90α. When cells were heated, CDC37 disassociated from HSP90α after CDK1 binding to HSP90α. In the absence of 17-DMAG, CDK1 could be refolded to a mature soluble state during the recovery phase. In contrast, in the presence of 17-DMAG, CDK1 could not bind to HSP90α, thus CDK1 could not progress on its folding and maturation pathway and partitioned into the insoluble fraction. Combination of heat shock and 17-DMAG resulted in a decrease of soluble CDK1 due to its instability and the loss of HSP90 chaperone function.

## Discussion

As HSP90 has been considered as an anti-cancer target for more than 20 years^15^, a series of HSP90 inhibitors have been discovered, including the first generation inhibitor Geldanamycin and its derivatives (17-DMAG, 17-AAG, etc.), and the second generation inhibitors STA-9090, NVP-AUY922^26^. However, activities of their metabolites are different. Geldanamycin derivatives are metabolized by CYP3A4, both the primary compounds and the main metabolites inhibit CYP3A4, thereby slowing down the degradation of Geldanamycin derivatives in the body^27^. In contrast, STA-9090 and its related compound NVP-AUY922 are excreted quickly in cells with high expression of the UDP glucuronosyltransferase 1A (UGT1A) gene cluster, whereas Geldanamycin derivatives are not^28^. Probably for this reason, STA-9090 does not work well in HCC patients^29^.

17-DMAG primarily inhibits HSP90 by binding to the ATP binding site in the N-terminal domain of HSP90^16^, but due to benzoquinone reduction and GSH conjugation in its metabolism^30^, it also causes ROS production. Moreover, heat shock also induces ROS generation and DSBs directly, though the underlying mechanism has not been fully understood^31^. Huh7 and HepG2 exhibited an increased level of intrinsic ROS stress as compared to L02 (Fig. 4a). Using this biochemical feature to develop a novel therapeutic strategies might preferentially kill cancer cells through ROS-mediated mechanisms^32^. Furthermore, HSP90 in tumour cells exhibits a higher affinity to HSP90 inhibitors^33^ that could explain why HCC cells are more sensitive to HSP90 inhibitors especially in combination with heat shock treatment.

In the p53 mutant human liver carcinoma cell line Huh7 the 17-DMAG/heat-shock combination treatment did not reduce cell viability and did not increase the apoptosis rate and G2/M arrest as prominently as in the case of the p53 wild type human liver carcinoma cell line HepG2. Since p53 mutant cells like Huh7 have always higher expression levels of antioxidants genes than p53 wild-type cells^34^, the ROS generation, induced by 17-DMAG/heat shock co-treatment, was more efficiently counteracted by the cellular redox buffering systems in these cells as compared to the p53 wild-type cells HepG2 (Fig. 4b). Thus less DNA double-strand damage was produced as indicated by reduced γH2AX (Fig. 4c). The p53 mutant phenotype in Huh7 cells is also related to p21 deficiency, and p21 prevents the activation of the CDK1-Cyclin B1 complex, explaining the lower proportion of G2/M arrest and decreased apoptosis rates in Huh7 cells after the co-treatment.

ROS and DSBs instantly activate the DNA damage response signalling pathway, which involves several HSP90 clients, in particular kinases and transcription factors, such as p53, DNA-PK, Chk1, Wee1, CDC25C, CDK1 and Cyclin B1 (http://www.picard.ch/downloads/HSP90interactors.pdf). On the other hand, client proteins could also be damaged by ROS-inducing conditions like heat shock or by ROS directly, compromising DNA damage repair; for example, CDK1 forms insoluble aggregates after heat shock^35^. Maturation and stability of most kinases need the interaction of HSP90 and its cochaperons (HSP70, HOP, CDC37, p23, etc.)^36^; in particular, CDC37 is necessary for recognition of kinase clients by HSP90^16^. Although CDC37 transiently stabilizes kinase clients, interaction with HSP90 is essential to prevent client aggregation and degradation^37^. Thus, inhibition of HSP90 promised to attenuate kinases that depend on the function of HSP90, even if CDC37 were unaltered by the treatment. Heat causes misfolding, aggregation and inactivation of many cellular proteins. Misfolded and aggregated client proteins need to be disaggregated and refolded by the Hsp70/Hsp110 system^38^,^39^ and depend on HSP90 for maturation, or they are degraded rapidly^40^. Since most cancer cells have defective G1 checkpoints, they depend on S and G2 checkpoints for survival from DNA damage^41^. In addition, G2/M transition requires the activation of the Cyclin B1-CDK1 complex. Co-treatment of heat shock and 17-DMAG induced more DNA damage, attenuated the interaction of HSP90 and CDC37 and inhibited HSP90 function which led to increased aggregation and thus decreased soluble CDK1. In addition, co-treatment of heat shock and 17-DMAG significantly lowered Cyclin B1 mRNA and protein. Hence, less CDK1-Cyclin B1 complex could be formed in cancer cells, leading to G2/M arrest and apoptosis, especially in HepG2 cells with less activated CDK1-Cyclin B1 complex. Similar cell viability results were obtained when heat shock was combined with STA-9090, a structurally unrelated HSP90 inhibitor (Fig. S3).

Due to inefficient blood flow through the newly formed immature blood vessels within solid tumours, including HCC, tumour cores usually experience hypoxic conditions, which induces HIF-1α, a trancription factor known to promote chemotherapy resistance by several different mechanism^42^,^43^. Hyperthermia also increases the blood flow in solid tumours^6^ and thereby may increase delivery of 17-DMAG to the poorly vascularized tumour cores. In combination with the increased ROS levels generated by hyperthermia, this might be the reason for the more significant decrease of Cyclin B1 and CDK1 in tumours co-treated with hyperthermia and 17-DMAG.

Hyperthermia can be practiced as whole body hyperthermia and local hyperthermia, and both of them show a well tolerance up to 6 h^44^. Though the earlier clinical trial results of hyperthermia alone presented diverse sensitivity in various tumours^45^, nowadays there appears to be a renewed interest, thanks to several randomized studies demonstrating that the improvements in treatment outcome by combined hyperthermia therapy^46^. More and more evidence supports thermal therapy increases the effectiveness of radiotherapy or chemotherapy treatment. Till now, 601 clinical trials, which are combined with different hyperthermia treatments, have been carried on www.clinicaltrial.gov. By itself, thermal therapy can also kill cancer cells^47^,^48^. Prostate cancer xenograft tumour sizes were about 60% of control group on day 30 after mice were heated at 42–43 °C for 10 min using gold nanorod^49^. Comparing with local hyperthermia, whole body thermal therapy is a systemic treatment, and whole-body fever-range thermal therapy can safely treat cancer cells wherever they are throughout the entire body^50^,^51^. The mechanism is still unclear but more evidence presents that the phenomena is not only related to the thermal stress the cancer cells encountered^47^,^48^, but also related to the regulation of whole body immunity^52^. Whole-body hyperthermia combined with HIPEC for the advanced gastric cancer also achieved an encouraging result, which showed 1-year survival rate was 38.5% compared to the chemotherapy alone group rate of 19%^53^. In this study, a 1.5-h whole body hyperthermia with or without 17-DMAG for 5 weeks exhibited a 70.3% and 37.9% tumour growth inhibition, respectively. This results suggest hyperthermia and 17-DMAG might be also feasible to HCC. By using HIPEC with 17-DMAG after cytoreductive surgery^53^, 17-DMAG-conjugated metal nanoparticle to heat tumours^6^, and 17-DMAG intravenous injection combined with heated by regional hyperthermia applicator^44^ are the methods of clinical application of the combination therapy against HCC.

Taken together, our study demonstrated that heat shock and 17-DMAG synergized in anti-proliferative treatment of HCC, both *in vitro* and *in vivo*, by inducing more ROS and DNA damage. In addition, 17-DMAG led to a decrease of mitosis promoting factor-related HSP90 client proteins, like CDK1, Cyclin B1, CDC25C, and triggered G2/M arrest and apoptosis. Notwithstanding, several open questions still remain. What is the reason for the decrease of Cyclin B1 mRNA during the co-treatment; decreased transcription or enhanced degradation? Is a whole body hyperthermia feasible when using this strategy in patients? Or could the tumour tissue be heated locally, for example by application of specific tumour-targeted gold nanoparticles? Nevertheless, our approach of combining hyperthermia with HSP90 inhibitors as a therapeutic strategy for treating HCC achieved satisfying results both *in vitro* and *in vivo*. Whether such a combination therapy can achieve similar therapeutic effects in patients remains to be explored in clinical trials.

## Materials and Methods

### *In vivo* experience

5-week old male BALB/c nude mice were obtained from Southern Medical University Laboratory Animal Centre. All procedures in this animal study protocol were approved by the Institutional Animal Care and Use Committee of Southern Medical University and carried out according to the principles of the NIH Guide for the Care and Use of Laboratory Animals. 5 × 10^6^ Huh7 cells in 200 μL DMEM were injected subcutaneously into the flanks of the mice. After 10 days, the mice were divided into four groups to accept treatment. Mice in the 17-DMAG group and the combination group were injected intraperitoneally with 17-DMAG (dissolved in saline) at a dose of 25 mg/kg three times a week, while mice in the control group and the hyperthermia group were injected intraperitoneally with saline. 2 h latter, mice in the hyperthermia group and the combination group were transferred into a 42 °C thermostatic cabinet and kept there for 1.5 h. The weights of the mice and the longest and shortest diameters of the xenograft tumours were measured before each treatment. After 5 weeks, all mice were sacrificed. Xenograft tumours were isolated. The tumour volume was calculated according to the following formula: longest diameter × (shortest diameter)^2^ × 0.5.

### Cell culture

Hepatic cell line L02^54^, hepatocellular carcinoma (HCC) cell lines Huh7 and HepG2^55^, hepatic cell line QGY-7703 and HCC cell line QSG-7701 which were isolated from the same hepatocellular carcinoma patient^56^, were purchased from the Type Culture Collection of the Chinese Academy of Sciences, Shanghai, China. All cells were cultured in Dulbecco’s Modified Eagle Medium (Gibco) containing 10% fetal bovine serum (Gibco), 100 μg/mL streptomycin sodium and 100 U/mL penicillin G sodium (MP Biomedicals) at 37 °C and 5% CO_2_.

### Cell treatment

17-DMAG (Selleck) was added into the medium before cells were transferred to another incubator, of which the temperature had been set to 42 °C, for the indicated time plus 15 min. The experiment scheme is shown in Fig. 2a.

### Cell viability

Cells were seeded into 96-well plates in 100 μL medium. 24 h after treatments the medium was replaced by fresh medium with 10% CCK-8 (Dojindo). Absorbance of 450 nm was measure after a 2-h incubation at 37 °C.

### Flow cytometry

For analysing the cell cycle, cells were trypsinized and fixed in 70% cold ethanol at −20 °C. Before performing flow cytometry, cells were washed 3 times in PBS and treated with a final concentration of 0.2 mg/mL aRNase and 50 μg/mL propidium iodide (PI) for 30 min at 37 °C and protected from light. For the analysis of apoptosis, cells were trypsinized without EDTA. Apoptotic cells were monitored using the Annexin V-FITC/PI apoptosis detection kit (Tianjin Sungene Biotech Co). For ROS detection, cells were incubated with 10 μM 2′,7′-Dichlorofluorescin diacetate (DCFH-DA) for 30 min at 37 °C, then trypsinized and resuspended in PBS. Flow cytometry was performed on the Guava easyCyte HT system (Millipore Corporation).

### Western blotting

Lysis buffer containing 20 mM PIPES (pH 7.0), 100 mM NaCl, 2 mM Na_3_VO_4_, 20 mM Na_2_MoO_4_, 1 mM MgCl_2_, 30 mM NaF, 1% Triton X-100 and protease inhibitors (Pierce) was used to lyse the cells and grind tissue. After centrifugation (14000 g) at 4 °C for 15 min (cells) or 30 min (tissue), the supernatant was collected and boiled with 5 × SDS-PAGE sample buffer. The pellets were resuspended in the same volume of 2 × SDS-PAGE sample buffer, sonicated on ice for 10s, centrifuged, and the supernatant collected as the insoluble protein fraction. Proteins were separated by SDS-PAGE and transferred to PVDF membranes. Primary antibodies: CDC37 (sc-13129), CDC25C (sc-327), Wee1 (sc-325), HSP70 (sc-69705) were from Santa Cruz Biotechnology, β-actin was from Tianjin Sungene Biotech Co, Cyclin B1 (#4138), CDK1 (#9112), p-CDK1 (Y15) (#9111), γ-H2AX (#9718), p21 (#2947), HSP90α (#8165), p-HSP90α (T5/T7) (#3488) were from Cell Signaling Technology, and p53 (A5761) was from Abclonal. IRDye secondary antibodies were from LI-COR Biosciences. Blots were scanned by Li-COR Odyssey infrared imaging system and analyzed by Image J 1.49 (NIH).

### Real-time quantitative RT-PCR

Total cell RNA was isolated using RNAiso (Takara). 500 ng of total RNA was used for reverse transcription with PrimeScrip RT reagent Kit (Takara). Real-time quantitative PCR was performed using SYBR Premix Ex Taq (Takara) with the following primer: Cyclin B1 (Forward: 5′-ACCAAAATACCTACTGGGTCGG-3′, Reverse: 5′-GCATGAACCGATCAATAATGG-3′), β-actin (Forward: 5′-TGGCACCACACCTTCTACAAT-3′, Reverse: 5′-AGAGGCGTACAGGGATAGCA-3′). All steps were performed as described in the user manual of the kit.

### Immunofluorescence

Cells were fixed using 4% paraformaldehyde for 10 min at room temperature and then washed 3 times with PBS. Cells were permeabilized by incubation in methanol at −20 °C for 15 min. Cells were blocked with 3% BSA, and incubated with mouse anti-Cyclin B1 antibodies (Santa Cruz Biotechnology, sc-245) at 4 °C overnight. Subsequently, cells were incubated with Alexa Fluor 555 donkey anti-mouse IgG (Life technologies) at room temperature for 2 h. Finally, the nuclei were stained with DAPI. Images were captured on an Olympus FV1000 Confocal Laser Scanning microscope.

### Co-Immunoprecipitation

Cells were lysed as described above. The supernatant was rotated end-over-end for 3 h at 4 °C with rat anti-HSP90α (Assay Designs, ADI-SPA-840) or non-specific rat IgG followed by additional 3 h rotation at 4 °C with protein G agarose beads (Santa Cruz Biotechnology, sc-2002). Beads were washed 3 times with fresh lysis buffer, and boiled with 2 × SDS-PAGE sample buffer.

### Statistical analysis

Data were analysed by one-way ANOVA or factorial design ANOVA followed least-significant difference post-hoc test for homogeneity of variance or Welch test followed Games-Howell post-hoc test for heterogeneity of variance for normally distributed data, and Kruskal-Wallis test and Mann-Whitney U test for skewed data. All analyses were performed in SPSS 19.0 (IBM). A *P* < 0.05 was considered statistically significant.

## Additional Information

**How to cite this article**: Huang, Z. *et al*. Hyperthermia enhances 17-DMAG efficacy in hepatocellular carcinoma cells with aggravated DNA damage and impaired G2/M transition. *Sci. Rep.* **6**, 38072; doi: 10.1038/srep38072 (2016).

**Publisher's note:** Springer Nature remains neutral with regard to jurisdictional claims in published maps and institutional affiliations.

## Supplementary Material

Supplementary Dataset
