# Supplementary material for: Hyperthermia enhances 17-DMAG efficacy in hepatocellular carcinoma cells with aggravated DNA damage and impaired G2/M transition
Source: Sci Rep. 2016 Dec 2;6:38072. doi: 10.1038/srep38072 (PMC5133462; doi:10.1038/srep38072)
Supplement: Supplementary Dataset [file srep38072-s1.doc]

**Hyperthermia enhances 17-DMAG efficacy in hepatocellular carcinoma cells with aggravated DNA damage and impaired G2/M transition**

Zhizhou Huang^1*^, Xueqiong Zhou^1*^, Yangfan He^1^, Xiangyu Ke^1^, Ying Wen^1^, Fei Zou^1#^, Xuemei Chen^1#^

^1^Department of Occupational Health and Medicine, School of Public Health, Southern Medical University, 1838 Guangzhou Road North, Guangzhou, 510515, China

* These authors contributed equally to this work.

#Author for correspondence

Xuemei Chen

Department of Occupational Health and Medicine, School of Public Health, Southern Medical University, 1838 Guangzhou Road North, Guangzhou, 510515, China

Tel: 86-20-61648471

Fax: 86-20-62789125

E-mail: cxmcsz@smu.edu.cn

Fei Zou

Department of Occupational Health and Medicine, School of Public Health, Southern Medical University, 1838 Guangzhou Road North, Guangzhou, 510515, China

Tel: 86-20-61648301

Fax: 86-20-61648324

E-mail: zfei@smu.edu.cn

**Supplementary material**

**Figure S1**


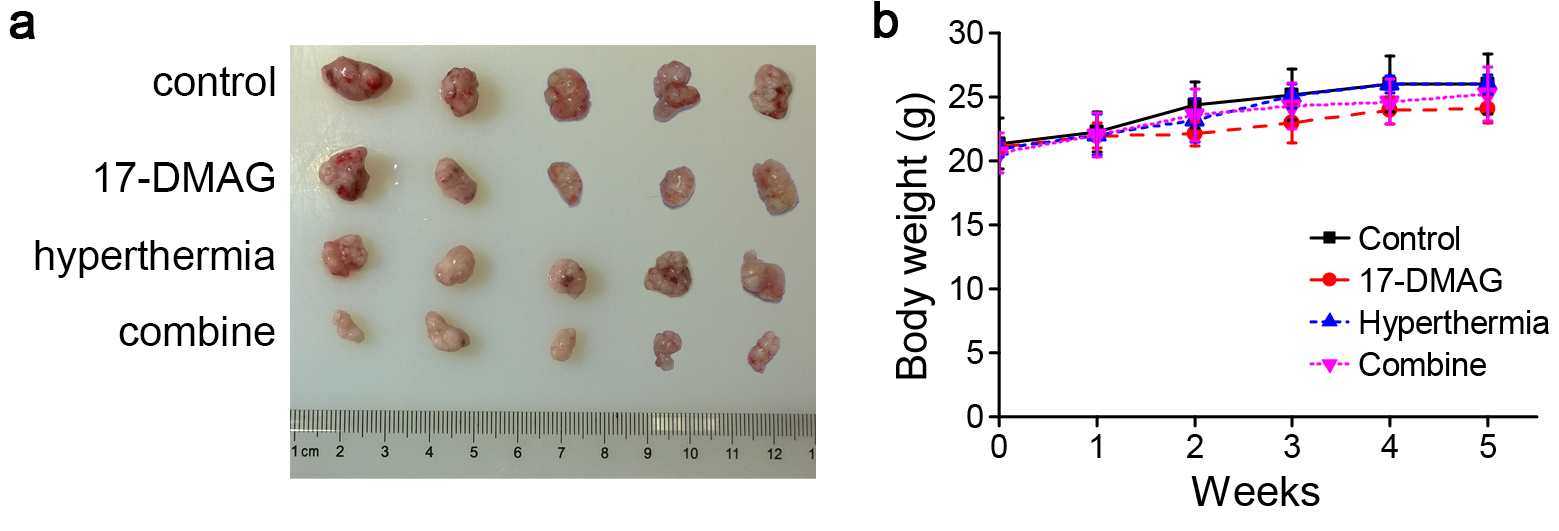


**Figure S1. Efficacy of hyperthermia and 17-DMAG co-treatment to xenograft tumours in nude mice.** 5×10^6^ Huh7 cells were injected into 5 weeks old male BALB/c nude mice. 10 days post injection mice with xenograft tumours were treated with a 1.5-h heat shock alone and/or 25 mg/kg 17-DMAG three times a week. (a) The resected tumours after the final treatment. (b) Changes of body weight during the course of the treatment.

**Figure S2**

**
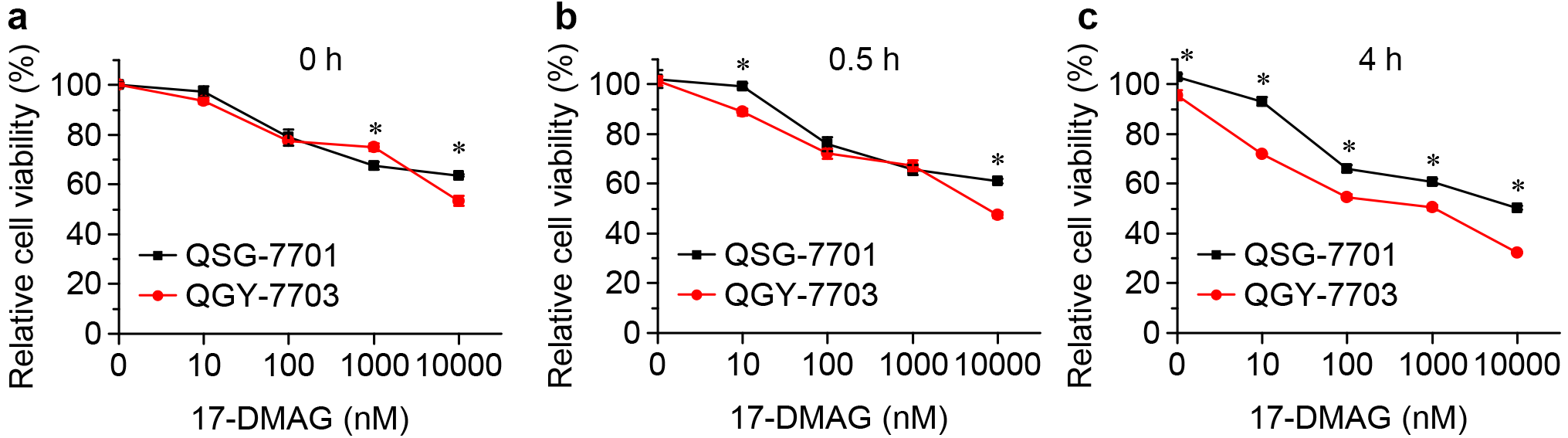
**

**Figure S2. Efficacy of heat shock and 17-DMAG co-treatment to HCC cells.** Human hepatocytes (QSG-7701) and its HCC cell (QGY-7703) from the same tumour were heated for 0 h (a), 0.5 h (b) and 4 h (c) with or without 17-DMAG at 42°C, then allowed to recover at 37°C until 24 h. Cell viability was detected by the CCK-8 assay. Results are shown as mean ± SD, n = 3. * *P* < 0.05 vs. QGY-7703.

**Figure S3**


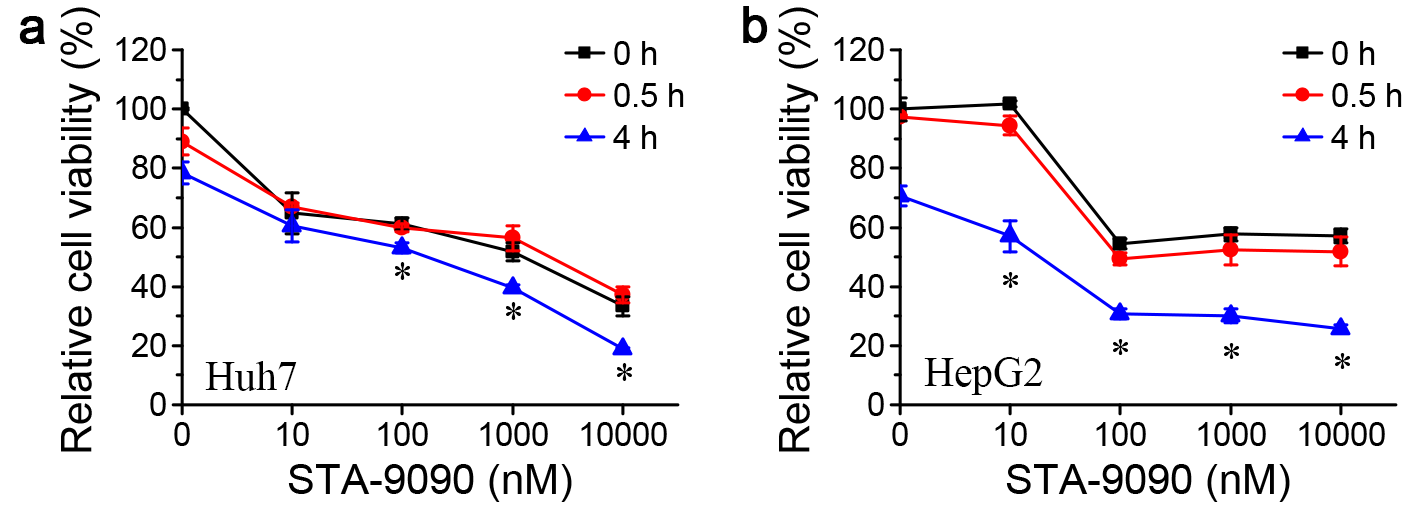


**Figure S3. Efficacy of heat shock and STA-9090 co-treatment to HCC cells.** Huh7 (a) and HepG2 (b) cells were heated for the indicated time with or without STA-9090 at 42°C, then allowed to recover at 37°C until 24 h. Cell viability was detected by the CCK-8 assay. Results are shown as mean ± SD, n = 3. * *P* < 0.05 vs. 0 h heat shock group.
